# Antineutrophil Cytoplasmic Antibody Vasculitis after Coronary Artery Bypass Grafting

**DOI:** 10.1155/2024/1212538

**Published:** 2024-08-12

**Authors:** Dwight D. Harris, Sharif A. Sabe, Afshin Ehsan

**Affiliations:** Division of Cardiothoracic Surgery Department of Surgery Cardiovascular Research Center, Rhode Island Hospital Rhode Island Hospital Alpert Medical School of Brown University, Providence, Rhode Island, USA

## Abstract

Antineutrophil cytoplasmic antibody (ANCA)-associated vasculitis is a group of rare autoimmune disorders associated with the presence of ANCA autoantibodies. We present the first reported case of acute ANCA-associated vasculitis following coronary artery bypass grafting in a 74-year-old male presenting on postoperative day 13 with shortness of breath, orthopnea, and acute kidney injury. Renal biopsy ultimately showed focal necrotizing and crescentic glomerulonephritis, and the patient was successfully managed with corticosteroids and outpatient rituximab. This rare case highlights the importance of having an expanded differential for uncommon causes of cardiovascular disease and unexpected outcomes after coronary artery bypass grafting.

## 1. Introduction

Antineutrophil cytoplasmic antibody (ANCA)-associated vasculitis (AAV) is a group of rare autoimmune disorders characterized by inflammation of small- to medium-sized blood vessels associated with the presence of ANCA autoantibodies [1]. The exact cause of AAV is not fully understood, but it is believed to involve a combination of genetic predisposition and environmental triggers resulting in autoantibodies to neutrophils [1, 2]. ANCA antibodies contribute to the damage of blood vessels, resulting in inflammation and tissue injury. Symptoms of AAV depend on the affected organs but may include joint and muscle pain, respiratory symptoms, hematuria, AKI, and dermal lesions [3]. Diagnosis of AAV involves a combination of clinical evaluation, the presence of serum inflammatory markers, ANCA antibody testing, imaging studies, and biopsy of affected tissues [3]. AAV has been shown to significantly increase cardiovascular risk [4, 5, 6]. Much of this increased risk is from steroid therapy; however, some studies suggest that AAV patients are at significant risk of cardiovascular event within the first year of diagnosis due to active disease [7, 8]. We present the first reported case of acute AAV diagnosed following coronary artery bypass grafting (CABG) in a patient presenting with shortness of breath, orthopnea, and AKI.

## 2. Case

The patient is a 74-year-old male that presented to an outside hospital with chest pain. He was found to have an ST segment elevation myocardial infarction and underwent cardiac catheterization which showed severe multivessel coronary artery disease. He was started on a heparin drip and transferred for surgical evaluation. He had no recent history of weight loss, arthralgias, or bleed. Echocardiogram demonstrated a normal ejection fraction and no valvular pathology, and his preoperative renal function was normal with a baseline plasma creatinine of 0.7–0.8 mg/dL. His workup did not show concern for atypical presentation such as coronaritis on angiography, myocarditis, pericardial effusion, or coronary artery aneurysms. He underwent a two-vessel CABG with a left internal thoracic artery and saphenous vein graft. His postoperative stay was complicated by atrial fibrillation requiring initiation of amiodarone and rivaroxaban. He was discharged home on postoperative day 7.

**Figure 1:**
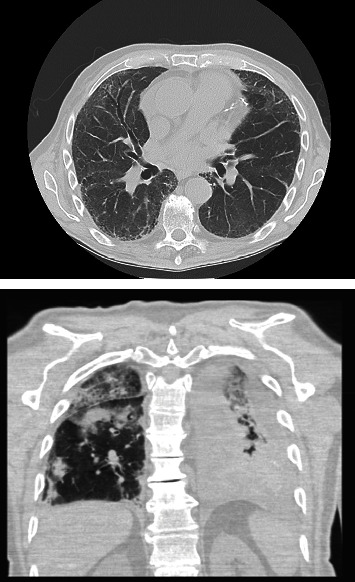
Computed tomography scan of the chest demonstrated multiple infiltrates and ground glass opacities.

He re-presented to the emergency room on postoperative day 13 with shortness of breath and orthopnea. He was placed on supplemental oxygen. Computed tomography scan of the chest demonstrated a left sided pleural effusion, small pericardial effusion, and multiple infiltrates concerning for pneumonia (Figure 1). He had a creatinine of 1.64 mg/dL which was a significant increase from his creatinine at discharge (0.83 mg/dL). He had a C-reactive protein 165 mg/L, pro-BNP 589 pg/mL from 470 pg/mL, white blood cell count of 22, hemoglobin of 7.2 g/dL from 7.5 g/dL, and negative blood cultures. He was started on antibiotics and admitted for further management. His shortness of breath was thought to be due to a combination of volume overload and pneumonia. Despite treatment with furosemide and antibiotics (piperacillin and tazobactam), he continued to worsen. Nephrology was consulted for his increasing creatinine, and pulmonology was consulted for the management of his shortness of breath. Given the lack of response to antibiotics and dieresis, the pulmonology service recommended assessment for an autoimmune process. He was subsequently noted to have elevations in his erythrocyte sedimentation rate, C-reactive protein, and myeloperoxidase antibody. Additionally, his ANCA antibody resulted as positive. The patient had negative PR3 ANCA, anti-GBM, ANA, and dsDNA antibodies. Based on these findings, the rheumatology service was consulted for further management. His creatinine peaked at 3.62 mg/dL on hospital day 9, and he was started on 60 mg/day prednisone. The patient's symptoms and creatinine improved with steroids, and a 10 mg per day taper was started per the rheumatology service. On hospital day 17, however, he had worsening respiratory symptoms. Repeat computed tomography scan showed worsening pulmonary infiltrates, and pulmonology and rheumatology recommended 1,000 mg of methylprednisolone for 2 days and increasing prednisone back to 60 mg/day. Given worsening of his pulmonary disease, the patient had a renal biopsy on hospital day 19. The biopsy ultimately showed mild background scarring (10%) and 9% sclerotic glomeruli consistent with ANCA glomerulonephritis. He improved with the increase in steroids and was discharged home on hospital day 24 on 60 mg/day prednisone. At the time of discharge, his creatine had improved to 1.10, and he was requiring 2 L nasal canula with ambulation. Immunosuppressive therapy with rituximab was initiated as outpatient therapy and prednisone reduce by 10 mg every 2 weeks under the direction of the rheumatology service.

## 3. Comments

We present the first reported case of AAV following coronary artery bypass grafting in a 74-year-old male who was readmitted with shortness of breath, orthopnea, and AKI. This case not only sheds light on a previously unreported occurrence; however, it is impossible to know if this represents undiagnosed AAV first presenting as coronary artery disease made worse by cardiac surgery or an extremely uncommon complications following cardiac surgery.

The mechanism for the development of AAV is not fully understood [2]. Trigger events have been described in the literature but are usually related to new medications or infections [9, 10]. There are no current reports in the literature of cardiac surgery as a potential trigger for the development of AAV. Cardiac surgery and bypass physiology have the potential to result in changes to vascular permeability and significant tissue injury throughout the body. It is possible that the stress and microscopic tissue injury form cardiac surgery resulted in the production of ANCA antibodies. However, given the lack of pre-op ANCA testing, it is impossible to exclude the possibility that the patient had preexisting subtle AAV and surgical stress created a favorable environment for the development of symptomatic AAV. AAV has been shown to significantly increase the life time risk of cardiovascular events and mortality [4, 5, 6, 7]. Given the lack of pre-op testing and the prevalence of cardiovascular disease in AAV patients, it is possible that the patient had AAV with CAD being an atypical initial presentation. The stress of cardiac surgery and bypass physiology could have pushed the patient into severally symptomatic AAV.

## 4. Conclusion

We present a case of the diagnosis of AAV following coronary artery bypass grafting. This rare case highlights the importance of having an expanded differential both during preoperative work-op and while managing late and/or atypical complications. Surgeons and cardiologists should bear in mind that CAD may be an early presentation of AAV, and when faced with a late presentation of AKI or pulmonary complications, cardiac surgeons should consider ANCA as a potential diagnosis in the absence of more traditional explanations.

## Data Availability

All data are available by request from the corresponding author.
